# Mitigating Effects of Nest Structure on Predation Risk in Hainan Partridge ( *Arborophila ardens* )

**DOI:** 10.1002/ece3.74158

**Published:** 2026-08-31

**Authors:** Yuhan Zhang, Yishuo Ding, Jing Wang, Qingling Zeng, Yuxin Xu, Wei Liang, Xiaodong Rao

**Affiliations:** ^1^ School of Tropical Agriculture and Forestry Hainan University Danzhou China; ^2^ State Key Laboratory of Animal Biodiversity Conservation and Integrated Pest Management, Institute of Zoology Chinese Academy of Sciences Beijing China; ^3^ Ministry of Education Key Laboratory for Ecology of Tropical Islands, Key Laboratory of Tropical Animal and Plant Ecology of Hainan Province, College of Life Sciences Hainan Normal University Haikou China; ^4^ Intelligent Forestry Key Laboratory of Haikou City Hainan University Haikou China

**Keywords:** *Arborophila ardens*, artificial nest, camera trap, nest predation, nest structure

## Abstract

Nest predation is the primary factor contributing to breeding failure, particularly in tropical regions. Enclosed nest structures can disrupt predator search efficiency and reduce predation risk. However, their effectiveness varies with environmental conditions. In this study, we tested whether the arched covering structure of Hainan Partridge ( *Arborophila ardens* ) nests reduces predation risk by disrupting predator search efficiency, and compared the difference in artificial nest predation between the interior and exterior of the national park. To evaluate this, we conducted artificial nest experiments with camera trapping from April to June 2025 across four breeding sites located both inside and outside the National Park of Hainan Tropical Rainforest on Hainan Island, south China. We deployed a total of 204 artificial nests containing domestic pigeon eggs, comprising equal numbers of covered nests designed to mimic the nest structure of the Hainan Partridge and uncovered control nests. Results showed that the predation rate in the uncovered group was significantly higher than that in the covered group (62.75% vs. 46.08%, *χ* ^2^ = 5.711, *p* = 0.017). However, covered nests exhibited varying degrees of anti‐predation effects within and outside the National Park of Hainan Tropical Rainforest. The primary predators were birds (Silver Pheasants *Lophura nycthemera* , Indochinese Green Magpies *Cissa hypoleuca* ) and rodents (in the family Muridae), followed by other mammalian predators (Wild Boars *Sus scrofa* , Rhesus Monkeys *Macaca mulatta* , Chinese Ferret‐badgers *Melogale moschata* ) and reptiles (Common Water Monitors *Varanus salvator* ). These findings highlight the functional limitations of nest‐structure evolution within complex predation environments. Through comparisons between the interior and exterior of the national park, we also reveal the impact of conservation management on nest predation risk in tropical pheasants, providing critical insights into the trade‐offs between reproductive strategies and survival pressures in tropical pheasants.

## Graphical abstract

Enclosed nest structures can disrupt predator search efficiency and reduce predation risk. Predation rate in the uncovered group was significantly higher than that in the covered group. Our study highlights the functional limitations of nest‐structure evolution within complex predation environments.

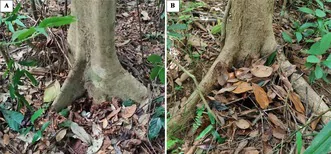

## 1 Introduction

Reproduction plays a vital role in avian life history (Martin 1987; Zheng 2012). Bird nests, as critical sites for offspring rearing, have long been central to evolutionary ecology research (Collias 1997; Fang et al. 2018; Hansell 2000; Mainwaring et al. 2023). Birds exhibit remarkable diversity in nest structure (Collias and Collias 1984; Mainwaring et al. 2023). Among these, the evolution of enclosed nest structures is generally considered an important adaptive response to specific environmental pressures, with domed nests among the primary forms (Collias 1997; Duursma et al. 2018; Mainwaring et al. 2023). The evolutionary history of domed nests originates from primitive passerines, such as Tyrannidae, Thamnophilidae, and the Furnariidae (Collias 1997; Hansell 2000). Research indicates they may have evolved from open cup nests (Collias 1997; Ocampo et al. 2023), potentially aiding offspring in withstanding adverse climatic conditions (Humphreys et al. 2007; Perez et al. 2020; Colombo et al. 2024). For example, Duursma et al. (2018) found that domed nests were more prevalent in arid regions than in non‐arid regions of Australia. In addition, domed nests are also thought to reduce the risk of offspring predation (Oniki 1985; Collias 1997; Hall et al. 2015).

Nest predation is a key factor affecting avian reproductive success, directly affecting population persistence and extinction (Martin 1992; Lima 2009). Numerous studies have shown that certain specialized nest structures can effectively reduce nest predation risk by blocking or disrupting predator search efficiency, thereby improving reproductive success (Collias and Collias 1984; Mainwaring et al. 2015). For instance, the nest of Yellow‐rumped Thornbill ( *Acanthiza chrysorrhoa* ) comprises a lower partially enclosed domed section and an upper open‐cup section; eggs are laid in the lower section, while the upper section may deceive predators (Galligan and Kleindorfer 2008). Nests of Penduline birds in the family Remizidae feature pouch‐like structures with narrow tubular entrances that effectively impede predator access (Zheng et al. 2018; Huang et al. 2025). Notably, the Cape Penduline Tit ( *Anthoscopus minutus* ) even constructs a closed false passage at the nest entrance, which serves to confuse and deter predators, such as snakes and rodents (Lloyd et al. 2017). Compared with open nests, enclosed nests, with their arched coverings, are believed to provide superior concealment for adults and offspring, thereby reducing visual detection by predators (Oniki 1985; Collias and Collias 1984; Wallace 1889). For example, Hall et al. (2015) conducted phylogenetic comparative analyses of nest height and structure among various Timaliidae species, revealing that domed nests were positioned closer to the ground than open‐cup nests. This observation implies that the domed nest structure may partially offset the high predation risk associated with ground nests. However, some studies have found no differences in predation rates between domed and open nests (Martin et al. 2017; Mouton and Martin 2019), with some domed nests even experiencing high predation. This observation suggests that the primary function of domed nests may be to protect against adverse weather conditions rather than to reduce predation risk (van Bael and Pruett‐Jones 2000; Hatchwell et al. 2013). These studies indicate that the effectiveness of domed nests in reducing predation risk varies by species, geographic location, and environmental conditions, underscoring the need for additional quantitative studies across a broader range of species to confirm their functional efficacy.

Pheasants (Phasianidae) exhibit high sensitivity to environmental changes, rendering their population dynamics effective indicators of environmental conditions (Zhang et al. 2003; Ahmad 2022). However, this sensitivity renders them susceptible to human disturbance, leading to heightened endangerment (Wang et al. 2017). Moreover, human disturbance may indirectly affect pheasant reproductive success by altering predator community composition and predation pressure (Beale and Monaghan 2004; Villalva et al. 2025). Therefore, understanding the reproductive strategies and nest predation factors influencing rare and endangered pheasants is crucial for their conservation and habitat management. Furthermore, most pheasants nest on the ground in open structures (Zheng 2015; Ding et al. 2019), a behavior that further heightens the vulnerability of their reproductive processes to disturbance or predation. In contrast to most pheasants that construct rudimentary open nests, certain *Arborophila* species use dead branches and fallen leaves from the nest site to form an arched dome covering over the nest body, a physical feature that substantially improves concealment (Rao et al. 2017; Fu et al. 2018). However, no studies have systematically evaluated the effectiveness of these nest structures in mitigating pheasant nest predation risk.

The Hainan Partridge ( *Arborophila ardens* ) (Galliformes, in the Phasianidae family) is endemic to Hainan Island and classified as a Class I National Key Protected Wild Animal in China. According to the 2025 IUCN Red List (HBW and BirdLife International 2025), this species is listed as globally Vulnerable. Hainan Partridges nest on the ground, and under natural conditions they only build arched nests completely covered by dry branches and fallen leaves with a lateral opening (Rao et al. 2017); no open nests have been found. Therefore, comparing predation rates across nest structure types provides a direct test of whether the covered structure confers an adaptive benefit by reducing predation risk in the Hainan Partridge. Strict protection management is enforced within the National Park of Hainan Tropical Rainforest, where human disturbance is low and the predator community is relatively rich. Outside the park, however, human activities have a greater impact and biodiversity is lower. Such environmental differences may lead to variations in nest predation rates and predator composition.

In this study, we compared nest predation rates between simulated open‐cup and enclosed‐arch artificial nests at four forest sites, two located inside and two outside the National Park of Hainan Tropical Rainforest, all within the distribution range of Hainan Partridges. Our aims were to: (1) assess the effectiveness of the arched covering structure in mitigating nest predation risk; (2) compare nest predation rates and predator composition between the interior and exterior of the national park to evaluate the actual conservation effectiveness of the national park for ground‐dwelling pheasants; and (3) use camera trapping to document potential nest predators of Hainan Partridges, reveal the main factors affecting nest predation rates, and provide a basis for conservation strategies for rare and endangered tropical rainforest pheasants.

## 2 Methods

### 2.1 Overview of the Study Area

**FIGURE 1:**
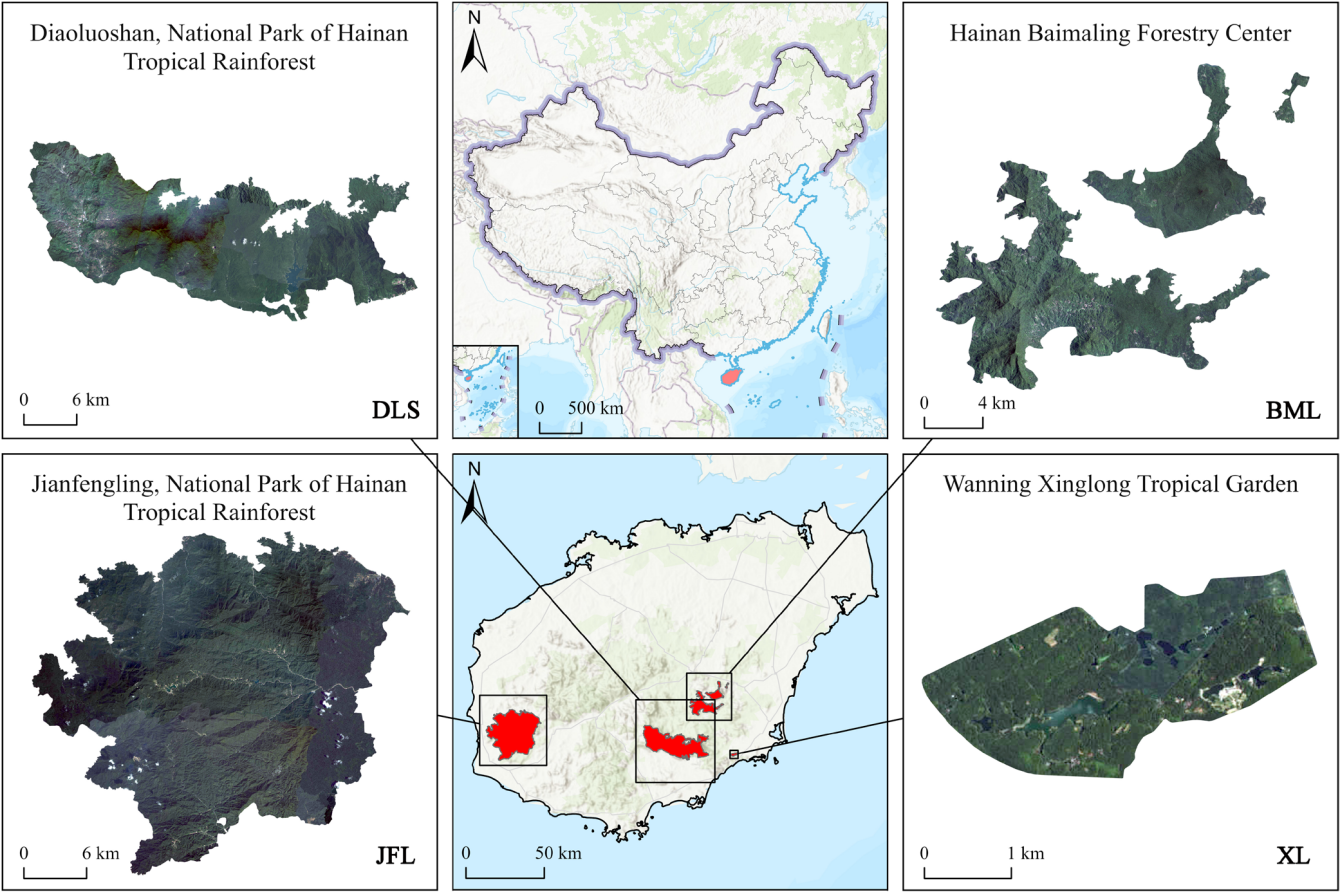
Distribution of the four study sites and satellite imagery.

Between April and June 2025, artificial nest experiments were conducted at four study sites: two sites located within National Park of Hainan Tropical Rainforest—Jianfengling (hereafter “JFL”) (108°44′–109°04′ E, 18°35′–18°52′ N) and Diaoluoshan (hereafter “DLS”) (109°40′–110°04′ E, 18°3′–18°51′ N)—and two sites located outside the park—Xinglong Tropical Garden in Wanning (hereafter “XL”) (110°22′ E, 18°69′ N) and Baimaling Forest Farm (hereafter “BML”) (193°59′–194°05′ E, 19°05′–19°21′ N) (Figure 1). Basic information for each study area is as follows:

JFL is located in the southwestern part of Hainan Island, bordering Dongfang City to the north and Ledong County to the west and east, with a total area of 678.43 km^2^ and an elevation ranging from 112.8 to 1412.5 m. JFL has a tropical monsoon climate, with warm weather year‐round and rainy summers; the average annual temperature is 24.5°C and the average annual precipitation is 2265.8 mm. The vegetation in this area includes tropical montane rainforest, evergreen monsoon forest, tropical northern marginal ravine rainforest, semi‐deciduous monsoon forest, montane mossy elfin forest, and savanna (Wu et al. 2025).

DLS is located in the southeastern part of Hainan Island, spanning the four cities and counties of Lingshui, Wanning, Baoting, and Qiongzhong, with a total area of approximately 447 km^2^ and an elevation ranging from 50 to 1499 m. DLS has a tropical marine monsoon climate with abundant rainfall; the average annual temperature is 24.4°C, and the annual precipitation ranges from 1870 to 2760 mm, which can exceed 2850 mm. The forest coverage rate reaches 96.26%, making it an important tropical rainforest area in China. The vegetation is distributed along the altitudinal gradient as tropical lowland rainforest, tropical montane rainforest, montane evergreen elfin forest, and shrubland (Yang et al. 2024).

XL is located within the Xinglong Overseas Chinese Farm in Wanning City, southeastern Hainan Province, covering a total area of approximately 4 km^2^ with an elevation ranging from 40 to 337.3 m (Chen and Pan 2008). It has a tropical monsoon climate, with an average annual temperature of 24.8°C and annual precipitation ranging from 1646.6 to 2691.2 mm. The garden is divided into several zones, and the vegetation in some areas is a reconstructed tropical rainforest following artificial ecological restoration.

BML is located at the junction of Huishan Town in Qionghai City and Zhongping Town in Qiongzhong County (193°59′ E–194°05′ E, 19°05′ N–19°21′ N), with a total area of approximately 42.6 km^2^ and an elevation ranging from 25 to 1270 m. Situated in central Hainan, BML has a tropical monsoon climate, with an average annual temperature of 23.3°C and an average annual precipitation of 3039.5 mm. The vegetation types in this area include tropical montane rainforest, tropical humid rainforest, secondary tropical rainforest, shrubland‐grassland mosaics, secondary forest mosaics, and grassland.

### 2.2 Artificial Nest Simulation Experiments

**FIGURE 2:**
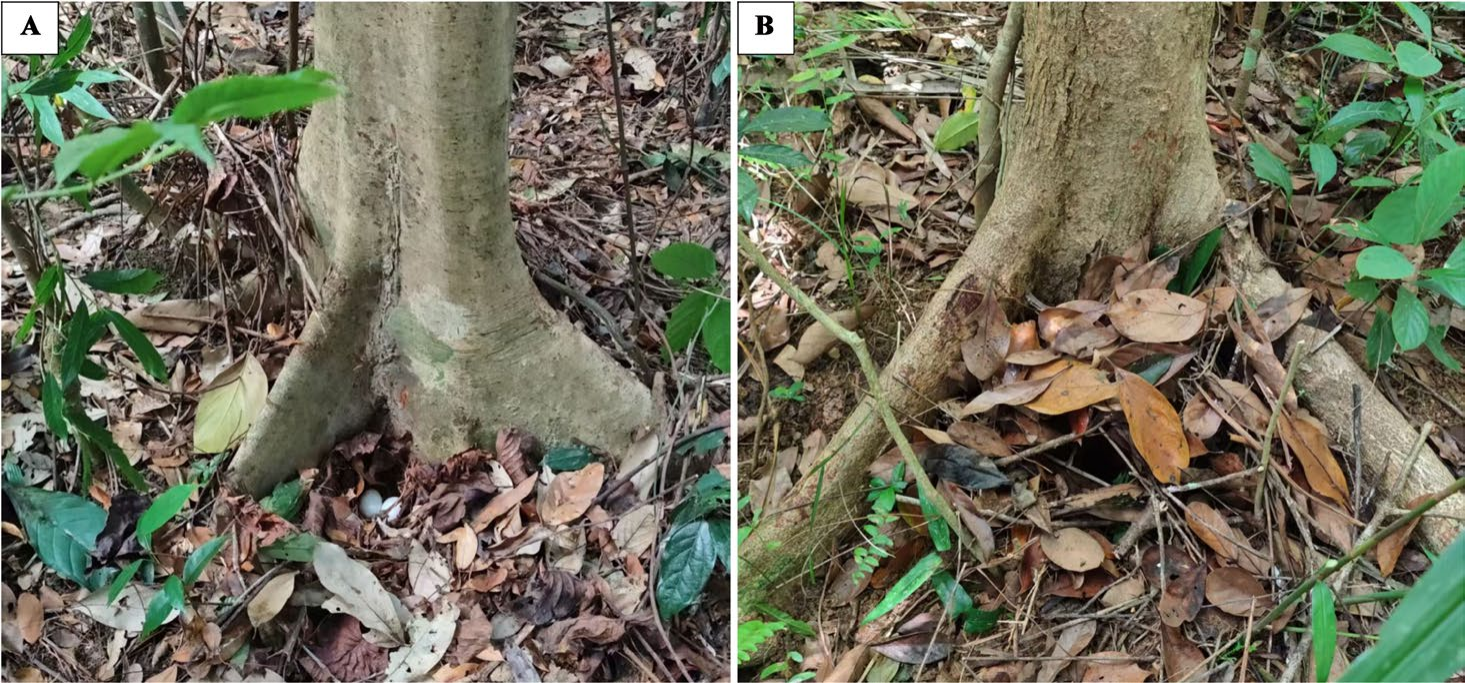
Photographs of artificial nests. (A) Not‐covered nest and (B) fully covered nest.

Currently, research on the nest‐site selection of Hainan Partridge is extremely limited; only Rao et al. (2017) reported that this species nests exclusively in natural forests. Therefore, in this study, we selected potential nest‐site areas based on the following criteria: (1) areas within natural forests showing clear signs of Hainan Partridge activity (especially foraging traces and feces) were chosen; (2) site locations with similar microhabitat characteristics were identified by integrating available nest‐site feature data (Zhang, Ding, Wang, Zeng, Xu and Rao, unpublished data) and the practical experience of local forest rangers. On this basis, we simulated the characteristics of natural Hainan Partridge nests by utilizing dead branches and fallen leaves from the environment, and artificial nests were subsequently deployed in potential nest site habitats across the four regions. A total of 204 artificial nests were deployed, encompassing 32 at XL, 60 at JFL, 60 at DLS, and 52 at BML. Covered and uncovered groups comprised half the sample size within each study site. Based on Hainan Partridge nest site characteristics, we placed artificial nests at the base of large trees, specifically between protruding buttress roots (Rao et al. 2017), with a minimum distance of 100 m between each nest. Based on egg color, size, and availability, we chose to use domestic pigeon (*Columba livia domestica*) eggs (41.15 ± 1.54 mm × 30.16 ± 0.94 mm; *N* = 20) as experimental eggs, which closely resemble natural Hainan Partridge eggs (35.82 ± 1.43 mm × 27.62 ± 0.96 mm; *N* = 4) (Rao et al. 2017). Two pure white domestic pigeon eggs were placed in each artificial nest, and two experimental treatments were established: (1) covered group, consisting of artificial nests simulating the arched covering structure of natural Hainan Partridge nests; (2) uncovered group, consisting of nests simulating only the natural nest cup with no covering material above (Figure 2). When deploying artificial nests, we treated one covered nest and one uncovered nest as a pair and placed them adjacently at sites with similar environmental conditions. This paired mixed arrangement ensured that covered and uncovered nests were interspersed, thereby minimizing the potential impact of differences in the nest surroundings on experimental results.

In light of insufficient breeding data for the Hainan Partridge, we referenced the natural incubation period of a closely related species with similar egg size, the Rufous‐throated Partridge ( *Arborophila rufogularis* ) (Ding et al. 2019), and set the experimental period at 21 days. The artificial nests were inspected on Days 10 and 21, with each nest photographed to document nest and egg conditions. The nests were reset following the first check. Instances of predation were recorded if at least one egg was found to be damaged or missing.

**FIGURE 3:**
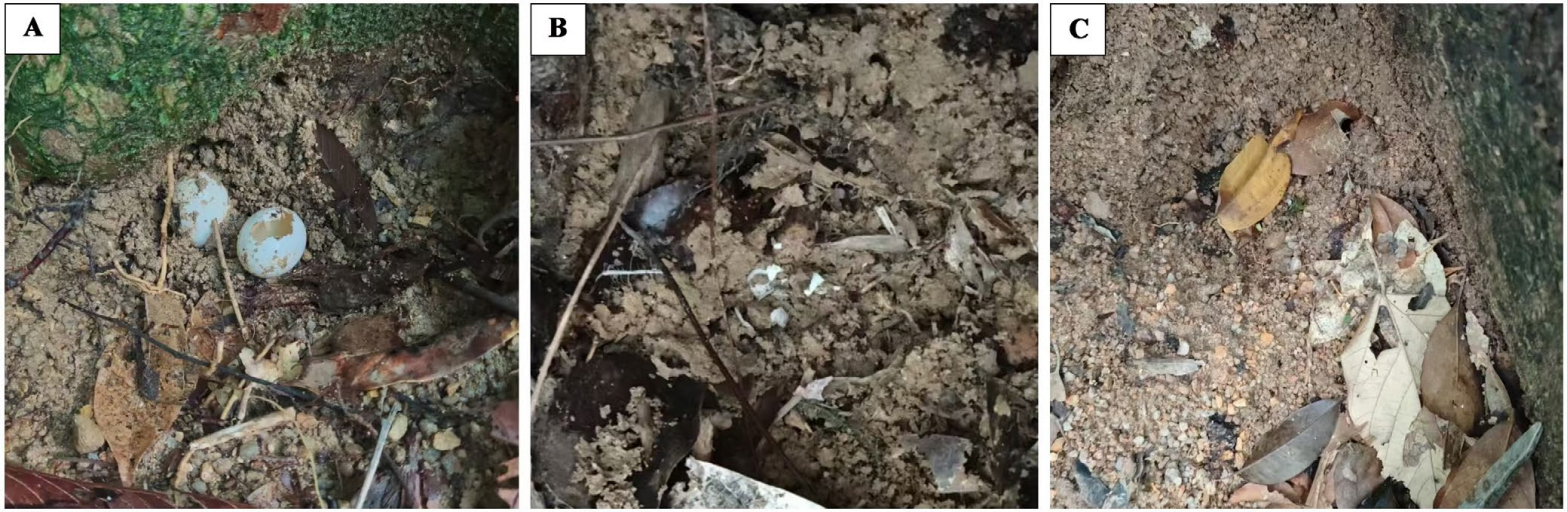
Photographs of different types of predation traces. (A) Type 1, where the eggshell is relatively intact with holes present; (B) Type 2, where the eggshell is fragmented, with less than half of the original shell remaining; and (C) no predation traces.

Of 204 artificial nests, 107 (52.5%) were monitored with camera traps (Ltl Acron: Ltl‐6630WMC) to document both primary and potential nest predators across the four study sites, encompassing 16/32 (50%) at XL, 29/56 (51.8%) at JFL, 29/60 (48.3%) at DLS, and 33/56 (58.9%) at BML. Cameras were configured for continuous recording throughout the day and night, with shooting mode set to “photo + video” and trigger sensitivity set to “medium” (based on Rao et al. 2023; Zeng et al. 2025). This resulted in the capture of three photographs and 30 s of video per activation. For predation events captured on camera, predator species and predation time were recorded. For predation events not recorded by cameras, predator types were inferred based on predation traces found within and around nests. Specifically, if the eggshell was relatively intact with holes, avian predation was inferred; if the eggshell was fragmented into pieces, mammalian predation was inferred; and if no trace remained, snake predation was inferred (Klug et al. 2010; Rao et al. 2023; Zeng et al. 2025). We classified post‐predation eggshell integrity into the following three types: (1) Type 1: the depredated egg retained more than half of a relatively intact eggshell, with varying numbers and sizes of holes on the shell (Figure 3A); (2) Type 2: only eggshell fragments of varying sizes remained, with the largest fragment not exceeding half of the entire egg (Figure 3B); (3) Type 3: no eggshell, fragments, or other related traces remained (Figure 3C). Since each nest contained two eggs, which could be preyed upon by different predators in separate events, for nests where both eggs were depredated, the predation traces of each egg were recorded separately. For Type 2 cases where eggshell fragments could not be distinguished as originating from one or two eggs, they were recorded as one egg belonging to Type 2 and the other as having ambiguous traces.

### 2.3 Data Processing and Analysis

Animals photographed by camera traps were primarily identified using the “Chinese Wildlife Manual of Mammals” (Smith and Xie 2009), “The CNG Field Guide to the Birds of China” (Liu and Cheng 2021), and “A Checklist on the Classification and Distribution of the Birds of China” (Fourth Edition) (Zheng 2023).

A binomial generalized linear model (GLM) with a logit link was used to examine the main effects and interaction of artificial nest location (inside vs. outside the national park) and Treatment (non‐covered nest, fully covered nest) on the response variable Result (Succeeded = 0, Failed = 1). The “car” package (v 3.1‐5; Fox et al. 2026) was used to perform Type II likelihood ratio tests to assess factor significance. Subsequently, the “emmeans” package (v 2.0.3; Lenth et al. 2026) was used for simple effects analysis, performing pairwise comparisons of predation rates between non‐covered and fully covered nests inside and outside the national park, with *p*‐values adjusted for multiple comparisons using the Tukey method. Odds ratios and their 95% confidence intervals were derived from back‐transformation of the log‐odds scale. Model overdispersion was diagnosed by the ratio of residual deviance to degrees of freedom. A chi‐squared test was used to analyze the significance of differences in overall predation rates among the four study sites. All analyses were performed in R 4.5.3 (R Core Team 2026), using packages including “readr” (v 2.2.0; Wickham, Hester, et al. 2026), “dplyr” (v 1.2.1; Wickham, François, et al. 2026), “car,” and “emmeans”.

## 3 Results

### 3.1 Artificial Nest Predation Rates

The predation results for covered and uncovered artificial nests at each study site were as follows: at JFL, 16 covered nests were depredated (53.33%) and 22 uncovered nests were depredated (73.33%); at DLS, five covered nests were depredated (16.67%) and 12 uncovered nests were depredated (40.00%); at XL, seven covered nests were depredated (43.75%) and eight uncovered nests were depredated (50.00%); at BML, 19 covered nests were depredated (73.08%) and 22 uncovered nests were depredated (84.60%). Overall, the predation rate of the uncovered group was higher than that of the covered group (62.7% vs. 46.0%), and the predation rate of artificial nests outside the national park was higher than that inside the park (66.7% vs. 45.8%). Among the four study sites, the artificial nest predation rate was highest at BML (78.85%), followed by JFL (63.33%), XL (46.88%), and DLS (28.33%).

In this study, the binomial generalized linear model included a total of 204 observations, of which 111 (54.4%) were predation events (coded as 1) and 93 (45.6%) were surviving nests (coded as 0). The model showed good overall fit, with no evidence of overdispersion (residual deviance/df = 1.33). The Type II likelihood ratio test indicated a significant main effect of Treatment (*χ* ^2^ = 6.00, df = 1, *p* = 0.0143) and a significant main effect of nest location (*χ* ^2^ = 9.02, df = 1, *p* = 0.0027). The Park × Treatment interaction was not significant (*χ* ^2^ = 0.58, df = 1, *p* = 0.4465), indicating that the effect of Treatment did not differ statistically across levels of park location.

Although the interaction was not significant, we conducted pre‐planned simple effects comparisons to test the differences between uncovered and covered nests inside and outside the national park. Within the national park (JFL and DLS), uncovered nests had a significantly higher predation rate than covered nests (OR = 2.43, 95% CI: 1.16–5.07, *p* = 0.0182). Outside the national park (XL and BML), uncovered nests also had a higher predation rate than covered nests, but the difference was not statistically significant (OR = 1.54, 95% CI: 0.62–3.84, *p* = 0.3558). In summary, although both nest location and Treatment had significant main effects overall, the higher predation rate of uncovered nests compared to covered nests was statistically significant only within the national park.

### 3.2 Nest and Potential Predators

Predator identity could only be confirmed with photo/video evidence for 17 of 111 predation events: avian predators, eight cases (47%); rodent predators, five cases (29%); other mammalian predators, three cases (18%); and reptilian predators, one case (6%) (Videos 1, 2, 3, 4, 5, 6, 7, 8, 9, 10, 11, 12, 13, 14, 15, 16, 17). Notably, at XL, none of the 15 failed nests were captured with predator images by cameras. The composition and numbers of nest predators captured on camera traps at each study site are shown in Table 1.

**VIDEO 1:** Eggs of the artificial nest were predated by a male silver pheasant in Baimaling Forest Farm. Video content can be viewed at https://onlinelibrary.wiley.com/doi/10.1002/ece3.74158.

**VIDEO 2:** Eggs of the artificial nest were predated by a male silver pheasant in National Park of Hainan Tropical Rainforest (Diaoluoshan). Video content can be viewed at https://onlinelibrary.wiley.com/doi/10.1002/ece3.74158.

**VIDEO 3:** Eggs of the artificial nest were predated by a female silver pheasant in National Park of Hainan Tropical Rainforest (Diaoluoshan). Video content can be viewed at https://onlinelibrary.wiley.com/doi/10.1002/ece3.74158.

**VIDEO 4:** Eggs of the artificial nest were predated by a female silver pheasant in National Park of Hainan Tropical Rainforest (Diaoluoshan). Video content can be viewed at https://onlinelibrary.wiley.com/doi/10.1002/ece3.74158.

**VIDEO 5:** Eggs of the artificial nest were predated by a female silver pheasant in National Park of Hainan Tropical Rainforest (Jianfengling). Video content can be viewed at https://onlinelibrary.wiley.com/doi/10.1002/ece3.74158.

**VIDEO 6:** Eggs of the artificial nest were predated by a female silver pheasant in National Park of Hainan Tropical Rainforest (Jianfengling). Video content can be viewed at https://onlinelibrary.wiley.com/doi/10.1002/ece3.74158.

**VIDEO 7:** Eggs of the artificial nest were predated by a female silver pheasant in the National Park of Hainan Tropical Rainforest (Jianfengling). Video content can be viewed at https://onlinelibrary.wiley.com/doi/10.1002/ece3.74158.

**VIDEO 8:** Eggs of the artificial nest were predated by a rhesus monkey in the National Park of Hainan Tropical Rainforest (Jianfengling). Video content can be viewed at https://onlinelibrary.wiley.com/doi/10.1002/ece3.74158.

**VIDEO 9:** Eggs of the artificial nest were predated by rodents in Baimaling Forest Farm. Video content can be viewed at https://onlinelibrary.wiley.com/doi/10.1002/ece3.74158.

**VIDEO 10:** Eggs of the artificial nest were predated by rodents in Baimaling Forest Farm. Video content can be viewed at https://onlinelibrary.wiley.com/doi/10.1002/ece3.74158.

**VIDEO 11:** Eggs of the artificial nest were predated by rodents in National Park of Hainan Tropical Rainforest (Diaoluoshan). Video content can be viewed at https://onlinelibrary.wiley.com/doi/10.1002/ece3.74158.

**VIDEO 12:** Eggs of the artificial nest were predated by rodents in National Park of Hainan Tropical Rainforest (Diaoluoshan). Video content can be viewed at https://onlinelibrary.wiley.com/doi/10.1002/ece3.74158.

**VIDEO 13:** Eggs of the artificial nest were predated by rodents in National Park of Hainan Tropical Rainforest (Diaoluoshan). Video content can be viewed at https://onlinelibrary.wiley.com/doi/10.1002/ece3.74158.

**VIDEO 14:** Eggs of the artificial nest were predated by a wild boar in the National Park of Hainan Tropical Rainforest (Jianfengling). Video content can be viewed at https://onlinelibrary.wiley.com/doi/10.1002/ece3.74158.

**VIDEO 15:** Eggs of the artificial nest were predated by an Indochinese green magpie in National Park of Hainan Tropical Rainforest (Jianfengling). Video content can be viewed at https://onlinelibrary.wiley.com/doi/10.1002/ece3.74158.

**VIDEO 16:** Eggs of the artificial nest were predated by a Chinese ferret‐badger in Baimaling Forest Farm. Video content can be viewed at https://onlinelibrary.wiley.com/doi/10.1002/ece3.74158.

**VIDEO 17:** Eggs of the artificial nest were predated by a common water monitor in the National Park of Hainan Tropical Rainforest (Jianfengling). Video content can be viewed at https://onlinelibrary.wiley.com/doi/10.1002/ece3.74158.

**TABLE 1:** Number of nest predation events recorded by camera traps across study sites.

Study area	Total predation events	Predator species	Number of events	Proportion within site (%)
Xinglong	0	—	0	—
Jianfengling	7	Silver Pheasant ( *Lophura nycthemera* )	3	42.9
		Rhesus Macaque ( *Macaca mulatta* )	1	14.3
		Wild Boar ( *Sus scrofa* )	1	14.3
		Indochinese Green Magpie ( *Cissa hypoleuca* )	1	14.3
		Common Water Monitor ( *Varanus salvator* )	1	14.3
Diaoluoshan	6	Silver Pheasant	3	50.0
		Rodents (Muridae)	3	50.0
Baimaling	4	Silver Pheasant	1	25.0
		Chinese Ferret‐badger ( *Melogale moschata* )	1	25.0
		Rodents	2	50.0
Total	17	—	17	—

According to the on‐site predation traces, at JFL, there were 17 eggs classified as Type 1 (28.8%), nine eggs as Type 2 (15.3%), 28 eggs as Type 3 (47.5%), and five eggs with ambiguous traces (8.5%). At DLS, there were 10 eggs classified as Type 1 (40.0%), two eggs as Type 2 (8.0%), 11 eggs as Type 3 (44.0%), and two eggs with ambiguous traces (8.0%). At XL, among the depredated eggs, there were 17 eggs of Type 1 (63.0%), three eggs of Type 2 (11.1%), six eggs of Type 3 (22.2%), and one egg with ambiguous traces (3.7%). At BML, there were 24 eggs of Type 1 (44.4%), nine eggs of Type 2 (16.7%), 21 eggs of Type 3 (38.9%), and zero eggs with ambiguous traces (0%).

**FIGURE 4:**
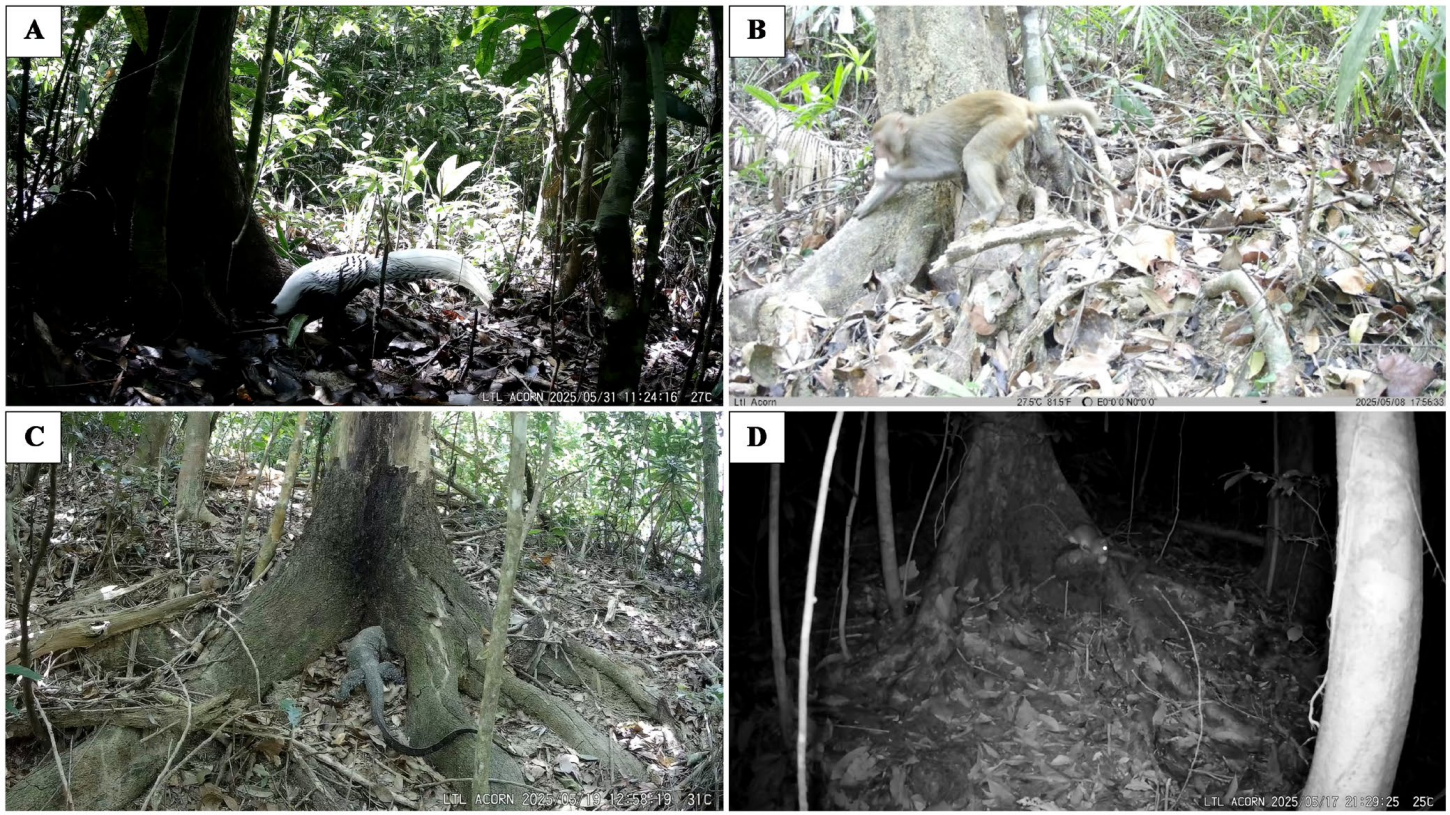
Examples of predators preying on eggs in artificial nests. (A) Silver Pheasant, (B) Rhesus Monkey, (C) Common Water Monitor, and (D) unidentified rodents (in the family Muridae).

Additionally, multiple nest‐visiting animal species were recorded across all study sites: JFL, 11 mammal and 14 bird species; DLS, 12 mammal and 20 bird species; XL, four mammal and nine bird species; and BML, 11 mammal and 13 bird species (Figure 4). Except for herbivorous and primarily invertebrate‐feeding species, such as some small passerines and sciurids, the remainder of these species was considered potential nest predators. The potential nest predators at each study site and their occurrence frequencies are shown in Table 2.

**TABLE 2:** Potential nest predators and their detection frequency using camera traps (number of sites) across study areas.

Study area	Potential predator species	Number of sites
Jianfengling	Silver Pheasant	25
	Northern Palm Civet ( *Paradoxurus hermaphroditus* )	11
	Wild Boar	9
	Rodents (Muridae)	5
	Red Junglefowl ( *Gallus gallus* )	3
	Chinese Ferret‐badger	2
	Rhesus Monkey	1
	Leopard Cat ( *Prionailurus bengalensis* )	1
	Greater Coucal ( *Centropus sinensis* )	1
Diaoluoshan	Rodents	19
	Silver Pheasant	13
	Wild Boar	10
	Northern Palm Civet	8
	Indochinese Green Magpie	3
	Chinese Ferret‐badger	2
	Leopard Cat	1
	Oriental Honey‐buzzard ( *Pernis ptilorhynchus* )	1
	Red Junglefowl	1
Xinglong	Rodents	4
	Northern Palm Civet	3
	Domestic Cat ( *Felis lybica* )	1
	Red Junglefowl	1
Baimaling	Rodents	34
	Silver Pheasant	12
	Northern Palm Civet	7
	Chinese Ferret‐badger	2
	Leopard Cat	2
	Indochinese Green Magpie	2
	Red Junglefowl	1

## 4 Discussion

This study found that the arched covering structure of Hainan Partridge nests mitigated the risk of nest predation to some extent. However, because dominant predator types and predation intensity differ across study sites across the island's tropical forest, the functional effectiveness of this specialized nest structure tends to vary geographically, but it cannot yet be confirmed that this effect differs distinctly between areas inside and outside the national park. Camera trapping revealed that the primary nest predators in the study sites were birds and rodents, followed by other mammalian predators and reptiles.

Enclosed nests are prevalent among various bird species (Hansell 2000; Ocampo et al. 2023). Early research has indicated that the primary ecological function of these structures is to provide defense against nest predators (Snow 1978; Oniki 1979, 1985; Collias and Collias 1984; Collias 1997). Within the genus *Arborophila*, arched covered nests have been documented in the Hainan Partridge and the Sichuan Partridge (Rao et al. 2017; Fu et al. 2018), but have not been reported in other congeneric species (Zheng 2015; Ding et al. 2019), suggesting that this structure may be a derived trait that evolved independently under specific environmental pressures. Both species inhabit high‐altitude, rainy and foggy subtropical or tropical forests, where such climatic conditions may favor leaf litter accumulation and the construction of covered nests, while high predation pressure may have driven the emergence of covered structures. This convergent evolution of the trait across different geographic regions warrants further verification through combined phylogenetic and paleoenvironmental data.

Nest concealment serves as an effective strategy for resisting predator searches, with multiple studies demonstrating a negative correlation between nest concealment intensity and nest predation rates (Hatchwell et al. 1996; Willson et al. 2001; Borgmann et al. 2013; Bu et al. 2019; Huge et al. 2024). Ground‐nesting groups generally use concealment strategies or camouflage, frequently nesting in densely vegetated areas (Albrecht and Klvaňa 2004; Li, Peng, et al. 2023), or by matching nest and egg coloration (Solis and de Lope 1995; Gómez et al. 2018; Alothyqi et al. 2024) and body coloration with the environment (Troscianko et al. 2016; Stevens et al. 2017). For example, the Japanese Quail ( *Coturnix japonica* ) selects environments that match egg coloration for laying whenever possible (Lovell et al. 2013; Alothyqi et al. 2024). Similarly, the Hainan Partridge employs concealment strategies in nest construction by incorporating dead branches and fallen leaves from the surrounding environment as nest covering, thereby effectively concealing nests within the environment (Rao et al. 2017). Combined with the domed covering structure, this is suspected to theoretically produce a strong mitigating effect on predation risk.

However, recent studies comparing multiple species have found no direct relationship between open or enclosed nest structures and predation risk, and instead suggest that their primary function may be to reduce disturbance to offspring by influencing abiotic factors (Mainwaring et al. 2014; Martin et al. 2017; Mouton and Martin 2019). Artificial nest experiments have also yielded inconsistent results: some studies have shown that covered nests can effectively reduce predation risk (Møller 1989; Jokimäki and Huhta 2000), while others have found no significant difference (Loiselle and Hoppes 1983; Zeng et al. 2025). In this study, artificial covered nests showed an overall defensive advantage, and the main effect model analysis indicated that the magnitude of this advantage did not differ between the interior and exterior of the national park. However, simple effects analysis revealed that covered nests exhibited a significant anti‐predation effect only within the national park, and thus their effectiveness appeared to be greater than in areas outside the park. This may be due to insufficient subsample size or statistical power outside the national park. Therefore, no definitive conclusion can yet be drawn as to whether the actual protective effectiveness of the arched covered nest of Hainan Partridge differs across different geographic regions. Particularly in tropical regions with high predation pressure, the defensive effectiveness of this nest structure may be relatively limited. Evaluating the defensive function of nest structures requires a comprehensive analysis that considers specific environmental contexts, predator community composition, and data sufficiency.

The primary reason for this substantial discrepancy between theoretical predictions and actual outcomes may stem from the different search strategies and perceptual modes employed by different predators in natural environments. This structure exhibits strong nest and egg concealment capabilities that can effectively interfere with avian predators primarily relying on visual searching (Møller 1989; Mouton and Martin 2019); however, for mammalian and reptilian predators that can locate nests using olfactory cues (Amo et al. 2004; Hughes et al. 2010; Li, Swerdloff, et al. 2023) or predators such as macaques and wild boars that search for prey through excavation across different habitats (Barrios‐Garcia and Ballari 2012; Kaisin et al. 2018), these structures substantially decrease interference with their search efficiency (Mouton and Martin 2019). This study inferred from camera trap monitoring that the main predators of open nests were birds, followed by mammals, whereas covered nests were more frequently preyed upon by mammals. This finding is consistent with the influence of predator perceptual strategies discussed above. However, a limitation here is that we clearly recorded only two predation events on covered nests (by a rodent and a Chinese ferret‐badger, respectively).

To further infer the predator guilds for nests where no predator was captured on camera, this study analyzed the depredated eggs from each site based on eggshell remains. Previous research has shown that avian predation is most likely to produce Type 1 predation traces, Type 2 traces are mostly caused by mammalian predation, and snake predation typically leaves no traces (Rao et al. 2023). A comparison of the trace composition across sites reveals that the predation trace type structures at JFL and DLS were relatively similar, both dominated by Type 3, with Type 1 also accounting for a relatively high proportion, suggesting that the predator guilds at these two sites may be dominated by snakes, followed by birds. At BML and XL, the proportion of Type 1 was the highest, and at XL, avian predation traces were overwhelmingly dominant; however, due to the small number of predation events at this site, the results should be interpreted with caution. According to the trace results, snakes and birds should be the main predator guilds. However, although the above trace‐based inferences are consistent with the composition of predator types directly recorded by camera traps in some respects, our camera trap recordings revealed that both birds and mammals were important predators (Figure 4), while reptilian predators were notably rare and no snake predation was recorded. Furthermore, in this study, of the eight nests with known mammalian predators, only one nest left Type 2 traces after predation. Additionally, among predation traces attributed to birds, two nests showed Type 2 and Type 3 traces. Camera traps recorded a rhesus monkey directly taking away a whole egg, and a rodent pushing a whole egg out. This demonstrates that inferring predator composition from predation traces has clear limitations.

Although trace analysis suggests that reptiles are the primary predator type for nests, predation trace identification itself involves uncertainty and inferential bias (Larivière 1999). Firstly, different predators may produce similar traces, and the same predator may also create different types of traces due to behavioral variation; for example, crows may feed by either directly carrying away eggs or destroying them on the spot (Montevecchi 1976; Bravo et al. 2020). Secondly, the time interval between predation occurrence and trace inspection may alter the original trace morphology due to factors such as rain wash, leaf litter, and soil burial, and microbial decomposition, thereby increasing the uncertainty of trace identification. Furthermore, secondary predation, in which the remains in a depredated nest attract other predators, may also cause changes in the traces (Stanton 1944). Therefore, future research requires increased camera trap deployment and coverage to further clarify the composition and behavioral patterns of primary Hainan Partridge nest predators.

This study revealed a trend that the protective effect of covered nests was higher inside the National Park of Hainan Tropical Rainforest than outside, which may stem from systematic variations in habitat quality, predator composition, and levels of anthropogenic disturbance between the interior and exterior of the national park (Rogan et al. 2022; Chen et al. 2023). National parks, as contiguous and strictly protected areas, not only effectively reduce human threats such as poaching (Lewis et al. 1990; Hou et al. 2024), but also provide intact, contiguous high‐quality habitats for wildlife such as pheasants (Chen et al. 2023), particularly supporting relatively abundant populations of the Silver Pheasant (Gao 1998; Yin et al. 2022). In this study, the Silver Pheasant was identified as one of the primary nest predators. As most birds primarily rely on visual cues when foraging (Fernández‐Juricic et al. 2004; Rubene et al. 2023), the Silver Pheasant, as a visually oriented predator, is susceptible to having its foraging efficiency disrupted by the concealment provided by nest structures. Furthermore, in this study, infrared camera monitoring results further confirmed that bird predators such as pheasants occur relatively frequently within the national park, particularly the Silver Pheasant, whose recording frequency was higher than at XL and BML outside the park, while mammalian predators (e.g., rodents, ferret‐badgers) were relatively scarce. This may explain the higher anti‐predation defensive efficacy within the park.

On the other hand, climate may also be a factor contributing to the regional differences in the defensive effectiveness of covered nests. In addition to being a key environmental factor influencing the composition and distribution of predator groups, thereby indirectly affecting nest predation rates (Cox et al. 2013), climatic factors can also directly influence the behavioral performance of predators (Gibbons and Semlitsch 1987; Dinsmore et al. 2002). For example, precipitation can limit the activity capacity of ectothermic animals, such as snakes, through temperature regulation, thereby reducing their foraging efficiency (Ford and Burghardt 1993; Peterson et al. 1993). Conversely, in humid environments, odors are retained and transmitted more effectively (Stauffer et al. 2011; Dreitz et al. 2012). Consequently, certain olfaction‐dependent predators may enhance their success rate in locating nests during rainfall (Webb et al. 2012). BML exhibits notably higher annual precipitation than that observed at the other three study sites (https://www.worldclim.org/), and this site supports various mammalian predators capable of locating prey through olfactory cues. This climatic factor may improve the efficiency of olfaction‐oriented predators by increasing the availability of olfactory cues in the environment. This may serve as an important mechanism leading to the relatively poor protective effectiveness of covered nests and the overall higher predation rates observed in this region. Therefore, although the covered nest structurally possesses visual concealment functions, its protective effectiveness did not demonstrate significant results in areas outside the park when confronted with predator communities dominated by mammals that primarily employ non‐visual foraging strategies.

In addition to predator composition, high predation risk environments may also be another important factor limiting the defensive function of covered nest structures. Tropical regions are characterized by high biodiversity and abundant predator types, with nest predation rates typically exceeding those in temperate and other regions (Robinson et al. 2000; Stutchbury and Morton 2022). Moreover, ground nests are more accessible to predators and often experience higher predation risk than that faced by other nest types (Manolis et al. 2002; Minias and Janiszewski 2023). Consequently, the interplay between high predation pressure in tropical regions and ground nesting strategies can substantially reduce the protective function of covered nest structures. In the DLS site, where predation rate was overall the lowest, this study observed that covered nests exhibited the most effective defense. This finding further corroborates the aforementioned viewpoint. Additionally, a larger nest body volume may contribute to covered nests experiencing higher‐than‐expected predation rates. Mouton and Martin (2019) pointed out that enclosed nests constructed by birds of similar body size were generally larger than open nests, with larger nests being more susceptible to detection by predators. A similar phenomenon was observed in this study: with similar nest cup dimensions, covered nests constructed to simulate the natural nest structure of Hainan Partridge, with additional dead branches and fallen leaves added as the arched covering, appeared visually larger than uncovered nests. Currently, although covered nest structures provide defensive advantages, their larger volumes may render them more susceptible to exposure, thereby offsetting some of their anti‐predation benefits.

However, our results may be influenced by the inherent limitations of the artificial nest experimental method. Although this method is widely used (Keyser et al. 1998; Krüger et al. 2018; Faria et al. 2022; Rao et al. 2023; Zeng et al. 2025), compared with natural nest‐based studies, its core limitation is the absence of parental birds (Burke et al. 2004; Thompson and Burhans 2004). Parental bird activity during incubation and brooding may expose nest locations, serving as important cues for predator nest searches (Bayne et al. 1997; Robel et al. 2003; Reidy et al. 2009). In addition, their defensive behaviors may directly influence predator success rates (Unzeta et al. 2020). Neglecting parental factors may result in biased estimates of actual nest predation risk. The tropical rainforest environment in which this experiment was conducted introduced further confounding factors: under frequent rainfall and dense vegetation, open nests without parental cleaning were easily covered by rain‐washed soil and fallen leaves, passively forming a concealed structure similar to that of covered nests. This led to structural convergence between the two nest types, confounding the true defensive effects and resulting in non‐significant differences in predation rates. Under natural conditions, with parental maintenance of nest structure, the mitigating effect of arched covered nests on predation risk is likely be greater than that indicated by our experimental findings.

This study, by comparing the predation rates of two types of artificial nests, verified that the arched covering nest structures of the Hainan Partridge possess certain anti‐predation functions. However, their effectiveness seems to be dependent on the predator composition: Within the national park, where visually oriented birds (such as the Silver Pheasant) are more prevalent, their concealment can effectively serve a defensive role. Conversely, in areas outside the park dominated by scent‐guided and burrowing mammals, the protective efficacy of this structure is relatively weaker. These findings emphasize that the evaluation of nest defense strategies must incorporate the composition and behavioral characteristics of local predator communities, but do not yet provide a context‐dependent explanation for the ongoing debate regarding the function of domed nests. Future research should integrate natural nest monitoring, predator behavior experiments, and multidimensional comparative studies with open nests of sympatric pheasant species, especially considering the relative abundance differences of the study species and major predators inside and outside protected areas, to systematically elucidate the adaptive significance of nest structures in changing environments and provide a scientific basis for pheasant conservation in diverse habitats.

## Author Contributions

**Yuhan Zhang:** data curation (equal), formal analysis (equal), investigation (equal), visualization (equal), writing – original draft (equal). **Yishuo Ding:** investigation (equal), methodology (equal). **Jing Wang:** investigation (equal), methodology (equal). **Qingling Zeng:** investigation (equal), methodology (equal). **Yuxin Xu:** investigation (equal), methodology (equal). **Wei Liang:** conceptualization (equal), supervision (equal), validation (equal), writing – review and editing (equal). **Xiaodong Rao:** conceptualization (equal), funding acquisition (equal), resources (equal), supervision (equal), validation (equal), writing – review and editing (equal).

## Funding

This work was supported by the National Natural Science Foundation of China (32360250), the specific research fund of The Innovation Platform for Academicians of Hainan Province (YSPTZX202408), the Hainan Institute of National Park and Hainan Province Science and Technology Special Fund (HINP, KY‐24ZK02, ZDYF2023RDYL01), and the Hainan Province Graduate Innovative Research Project (Qhys2024‐157).

## Disclosure

Declaration of Generative AI and AI‐Assisted Technologies in the Writing Process: We declare that in the process of writing this article, no generative artificial intelligence or artificial intelligence assisted technology was used.

## Ethics Statement

The experimental eggs used in this study are all unfertilized eggs, and there are no animal ethical issues.

## Conflicts of Interest

The authors declare no conflicts of interest.

## Data Availability

Data of artificial nest experiments, all R code used and 17 nest predation events recorded (Videos 1, 2, 3, 4, 5, 6, 7, 8, 9, 10, 11, 12, 13, 14, 15, 16, 17) for this study are publicly available in the Figshare repository at https://figshare.com/s/aafb9081efa2056ab31f (doi: https://doi.org/10.6084/m9.figshare.31035007).
